# Transglutaminase-Cross-Linked Tofu Suppressed Soybean-Induced Allergic Reactions by Enhancing Intestinal Mucosa Immune Tolerance

**DOI:** 10.3390/foods13081206

**Published:** 2024-04-16

**Authors:** Jing Bai, Yiling Zhou, Xinlei Xia, Zhihua Wu, Xin Li, Ping Tong, Anshu Yang, Hongbing Chen

**Affiliations:** 1 State Key Laboratory of Food Science and Resources, Nanchang University, Nanjing Dong Lu 235, Nanchang 330047, China; jiebenbai@163.com (J.B.); zhouyiling11111@163.com (Y.Z.); rose2jumping@163.com (X.X.); wuzhihua@ncu.edu.cn (Z.W.); zhizilixin@ncu.edu.cn (X.L.); tongping@ncu.edu.cn (P.T.); chenhongbing@ncu.edu.cn (H.C.); 2 School of Food Science and Technology, Nanchang University, Nanchang 330047, China; 3 Sino-German Joint Research Institute, Nanchang University, Nanjing Dong Lu 235, Nanchang 330047, China

**Keywords:** tofu, enzymatic cross-linking, mouse model, soybean allergy, intestinal mucosal immunity

## Abstract

Currently, food allergies are closely related to intestinal health, and ensuring the integrity and health of intestinal mucosa could reduce the incidence of food allergies. In this study, a soybean-allergic mouse model was used to explore the mechanism of intestinal mucosa immune response induced by enzyme-cross-linked tofu. The effects of enzyme-cross-linked tofu on intestinal mucosal immunity in mice were determined by hematoxylin–eosin (HE) staining and flow cytometry. Our results reveled that the MTG-cross-linked tofu reduced the reactivity of the intestinal mucosal immune system, which mainly manifested as a decrease in the dendritic cell (DC) levels of mesenteric lymph nodes (MLNs), increasing the Th1 cells and Tregs in Peyer’s patch (PP) nodes and MLNs, and inhibiting the Th2 cells. Compared with soy protein, enzyme-cross-linked tofu had less damage to the small intestinal tract of mice. Therefore, the above-mentioned results fully revealed that the enzyme-cross-linked tofu promoted the transformation of intestinal mucosal immune cells, shifted the Th1/Th2 balance toward Th1, and reduced its sensitization effect.

## 1. Introduction

Tofu is a gelatinous product with a three-dimensional meshwork structure that is formed by adding a dose of coagulant to cooked soy milk. It has a high nutritional value and is popular with consumers [1]. Tofu is made from soybeans, and the main ingredient is soy protein, so the composition and processing of soy protein is key to the quality of tofu. Soybean contains a variety of proteins. Currently, at least 16 kinds of allergenic proteins detected in soybean have been found to have binding properties with immunoglobulin E (IgE) in serum of soybean-allergic patients [2]. Glycinin (11S) and β-conglycinin (7S) are the two main allergenic proteins in soybeans [3]. It has been found that these two allergenic proteins are also the main components of tofu gel [4,5]. There is a high content of lysine and glutamine in soybean protein, so they are good cross-linking substrates for MTG [6]. It reported that MTG could improve the strength of tofu gel, as well as the molding and quality of tofu [7]. Due to steric hindrance, MTG-cross-linked proteins are also closely related to the molecular conformation of the substrate. Therefore, some sterically tight proteins can be better used as substrates for MTG after proper denaturation. Yasir et al. [8] found that MTG was easy to cross-link with proteins due to the unfolding of denatured protein molecules after soybean milk was boiled. In addition, the structure of the allergen is the material basis for its allergenicity and the allergenicity of tofu inevitably varies with the changes in the soy protein allergens during the formation of tofu. Therefore, the changes in the structure of soy glycinin and β-conglycinin are the key to modulating the allergenicity of tofu [9,10].

Allergenic proteins are digested in the gastrointestinal tract into polypeptides that cross the intestinal epithelial barrier and enter the intestinal mucosa, where they encounter relevant immune cells, triggering a series of intestinal mucosa immune responses [11,12]. A large number of dendritic cells (DCs), CD4+ T cells (Th1, Th2), CD8+ T cells, and mucosal Tregs are distributed in the lamina propria of the mucosa [13]. Peyer’s patches (PPs) and mesenteric lymph nodes are the main sites in the intestinal mucosa that recognize antigens and activate the immune cells [14,15]. Meng et al. [16] demonstrated that gavage with ALA (alpha-linolenic acid)-interacted BLA (alpha-lactalbumin) and BLG (beta-lactalbumin) promoted systemic allergic responses in mice and accelerated a shift in the Th1/Th2 balance toward a Th2 immune response as well as decreasing the number of Treg cells.

Enzymatic cross-linking of proteins is considered to be the most promising processing method to reduce their allergenicity [17,18]. Transglutaminases can alter the primary and advanced structure of proteins, including catalyzing the cross-linking within or between protein molecules, promoting the cross-linking between proteins and amino acids, and accelerating the hydrolysis of glutamine groups within protein molecules [7,19]. According to reports, transglutaminase (TG) processing reduced the allergenicity of peanut proteins [20]. It was shown that TG-cross-linking reduced the IgE binding of milk proteins and also decreased the secretion of Th2 cytokines in the splenocytes of milk-sensitized mice [21]. In the study of RBL-2H3 and KU812 cell models, it was found that enzymatic cross-linking of shrimp tropomyosin significantly inhibited the release of allergic mediators and cytokines. Meanwhile, the allergic responses in a mouse model could be alleviated by modulating the balance of Th1/Th2 immune cells [22]. A previous study showed that tropomyosin cross-linked with horseradish peroxidase induced oral tolerance and suppressed allergenicity in mice by reducing serum IgE and IgG1 levels, T-cell cytokine secretion, and the percentage composition of DCs [23]. We have also previously verified that enzymatic cross-linked tofu significantly reduced systemic allergic reactions in mice [24].

Therefore, the main purpose of this study is to investigate the mechanism of intestinal mucosa immune tolerance in allergic mouse model induced by enzyme-cross-linked tofu, including the antigen-presenting role of dendritic cells (DCs), the differentiation ability of CD4+CD8+ cells, the Th1/Th2 differentiation balance, and the regulation of Tregs. The results of this study are expected to facilitate the evolution of practical strategies for hypoallergenic enzyme-cross-linked soybean products.

## 2. Materials and Methods

### 2.1. Materials

The soybean ‘Dongnong 42’ was provided by the Northeast Agricultural University. MTG (food additive, enzyme activity: 102.3 U/mL) was provided by Taixing Yiming Biological Products Co., Ltd. (Taixing, China). The anti-mouse CD11c−FITC, anti-mouse MHCII−FITC, anti-mouse CD4−APC, anti-mouse CD25−PE, anti-mouse IL-4−BV421, anti-mouse IFN-γ−Percp, and anti-mouse Foxp3−APC were also purchased from eBioscience, Inc. (San Diego, CA, USA). All reagents were of analytical grade.

### 2.2. Preparation of Samples

The preparation of tofu with a single coagulant and a compound coagulant was carried out on the basis of a previously reported method [25]. Soybeans were soaked overnight and pulped at a ratio of 1:6.5 (*w*/*v*). Then, the raw soymilk was boiled (75 °C, 5 min; 95 °C, 10 min), and the pH was adjusted to 6.0. When the temperature of the soymilk was reduced to about 80 °C, gluconic acid lactone (0.33%) was added and left for 30 min at this temperature. Finally, the soymilk was transferred to a tofu mold and pressed for 3 h.

The production process for single MTG tofu is slightly different. The temperature of soymilk should be reduced to 50 °C and equilibrated for 10 min. The MTG (5 U/g pro) was added and placed in 50 °C for 3 h. Then, the enzyme inactivation process was carried out at a temperature of 90 °C for 5 min.

The composite coagulant tofu preparation: 0.33% gluconic acid lactone was added after enzyme inactivation, and the other steps were consistent with the preparation of single MTG tofu.

### 2.3. Mice

The specific-pathogen-free female BALB/c mice (SPF, 4–6 weeks of age) were purchased from Hunan Slake Jingda Laboratory Animal Co., Ltd. (Changsha, China) and all mice were cared for in accordance with the Guidelines for the Care and Use of Laboratory Animals published by the U.S. National Institutes of Health (NIH Publication 85-23, 1996). The experimental conditions were based on our previously reported method [24].

### 2.4. Experimental Design

The sensitization of the mice was carried out according to the method of Yang et al. [26] with slight modifications. All mice were divided into five groups (*n* = 8) randomly and assigned to a negative control group (PBS), a soybean-protein-positive group (control), a gluconic acid lactone tofu group (GDL), an enzymatic cross-linked tofu group (MTG), or a composite coagulant tofu group (GDL−MTG). All groups had intragastric administration once a week.

**Figure 1:**
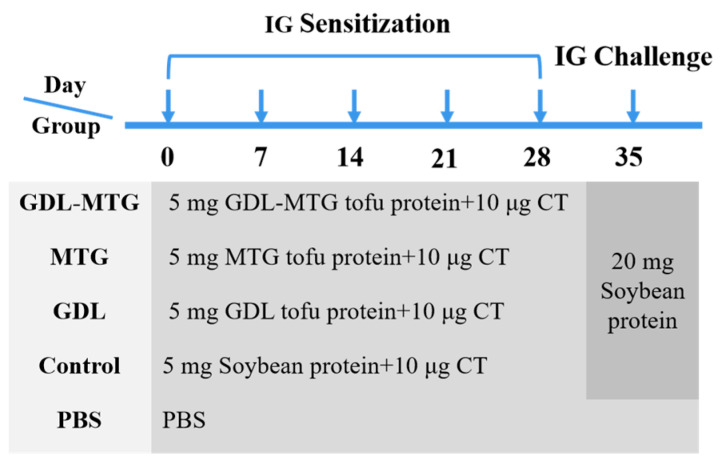
Experimental scheme of in vivo sensitization and challenge of BALB/c mice.

On days 0–28, mice in the PBS group were administered 0.3 mL PBS by oral gavage. Mice in the other groups were administered 5 mg protein solution or tofu solution containing 5 mg protein with 10 μg CT. On the 35th day, the mice in the experimental groups were orally challenged by gavage with 20 mg soybean protein solution except for the mice in the PBS group (Figure 1).

After the fifth gavage, the clinical symptoms of allergy in mice were observed and scored according to a previously reported method [2] (Table 1). Body weight and temperature in the mice were measured. All the mice were euthanized by dislocation.

### 2.5. Analysis of Immunoglobulin E (IgE)

IgE levels were detected by an enzyme-linked immunosorbent assay (ELISA) in accordance with the previously published method [26].

### 2.6. Preparation of PP and MLN Cell Suspension

The dislocated mice were immersed in sterile alcohol for 5 min. PPs and MLNs were isolated under aseptic conditions and homogenized in a 75 µm filter membrane by adding 10 mL of RPMI−1640. The filtered cells were washed twice and counted using a cell counter, and the cell concentration was adjusted to 4 × 10^6^ cells/mL.

### 2.7. Flow Cytometric Analysis of Immune Cells

#### 2.7.1. Detection of DCs

Acquired fresh MLN single-cell suspensions were added to the 96-well plate (100 μL/well, 4 × 10^6^/mL). The anti-mouse CD11c−APC and anti-mouse MHC−FITC or their isotype controls were added and incubated for 30 min in the dark. After the supernatant was discarded by centrifugation, the single cells were resuspended in 300 μL flow buffer and DC cells were analyzed using flow cytometry (BD, Franklin Lakes, NJ, USA).

#### 2.7.2. Detection of T Lymphocyte Subsets

Acquired fresh MLN single-cell suspensions were added to the 96-well plate (100 μL/well, 4 × 10^6^/mL). The anti-mouse CD4−FITC and anti-mouse CD3−APC and anti-mouse CD8−PE, or their isotype controls, were added and incubated for 30 min in the dark. After the supernatant was discarded by centrifugation, the single cells were resuspended in 300 μL flow buffer and the helper T cells (CD4+ and CD8+) were analyzed using flow cytometry (BD, USA).

Acquired fresh PP and MLN single-cell suspensions were added to the 96-well plate, respectively (100 μL/well, 4 × 10^6^/mL). The anti-mouse CD4−FITC and anti-mouse CD25−PE, or their isotype controls, were added and incubated for 30 min in the dark. After fixation and permeabilization, a flow buffer was added to the cells and incubated for 30 min in the dark. After the supernatant was discarded by centrifugation, the anti-mouse IFN-γ−Percp, anti-mouse IL-4−BV421, and anti-mouse Foxp3−APC were added and incubated for 30 min in the dark. The single cells were resuspended in 300 μL flow buffer and the Th1/Th2/Treg cells were analyzed through flow cytometry (BD, USA).

### 2.8. HE

The intestines of the mice (including duodenum, jejunum, and ileum) were isolated under aseptic conditions and preserved in tissue fixative (4% paraformaldehyde). The intestinal tissues were sequentially stained with hematoxylin–eosin. After dehydration, the intestinal tissues were embedded in paraffin and sectioned (4–5 μm). The pathological changes in the mouse intestines were observed and photographed with a CKX41 inverted fluorescence microscope.

### 2.9. Statistical Analysis

Immune cells were detected using a flow cytometer (BD AccuriTM C6 Plus, BD, Franklin Lakes, NJ, USA), and flow-cytometry-related data were analyzed using FlowJo software version 10.0. Charts were drawn using Origin 9.1 software and statistical analysis using SPSS 20.0 software. A one-way ANOVA analysis of variance was conducted, and differences between the sample means were analyzed. *p* < 0.05 was considered statistically significant.

## 3. Results

### 3.1. Evaluation of Allergic Mouse Model

**Figure 2:**
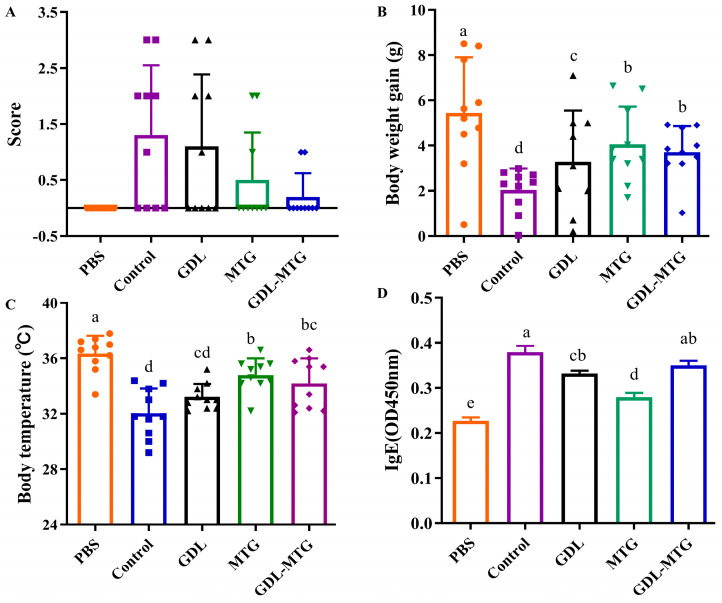
Allergic mouse model of enzymatic cross-linked tofu: (**A**) the score of anaphylaxis symptoms; (**B**) body weight gain; (**C**) body temperature; (**D**) levels of IgE. Control: soybean protein; GDL: gluconic acid lactone tofu; MTG: microbial transglutaminase-cross-linked tofu; GDL−MTG: composite coagulant tofu. Different letters on the histogram indicate a significant difference between the groups (*p* < 0.05).

The allergenicity of enzymatic cross-linked tofu in vivo was investigated using a mouse model induced by soybean protein (Figure 2). According to Table 1, no abnormalities were observed in the mice of the PBS group. In contrast, the mice in the soybean protein group (control) showed obvious allergic clinical symptoms, including pillar erection, shortness of breath, diarrhea, and reduced activity (Figure 2A). Mild allergy symptoms were observed in the mice of the MTG and GDL-MTG groups. Figure 2B shows body weight gain throughout the whole experiment. The weight gain in the mice of the PBS group was the largest, while the mice in the control group were the smallest. The mice in the other three groups weighed more than those in the control group but less than those in the PBS group. In Figure 2C, the body temperature of the mice in the control group significantly decreased (*p* < 0.05) after challenge by soybean protein, whereas the temperature of the mice in the GDL, MTG, and GDL−MTG groups was higher than that of the control group. Compared with the control group, the temperature of the MTG and GDL-MTG groups significantly increased (*p* < 0.05). As IgE is the main clinical indicator for allergy, the serum IgE levels were determined. In comparison with the PBS group, the levels of IgE were increased significantly in the control group, whereas those in the other three groups were slightly decreased (Figure 2D).

### 3.2. Role of DC Presentation

**Figure 3:**
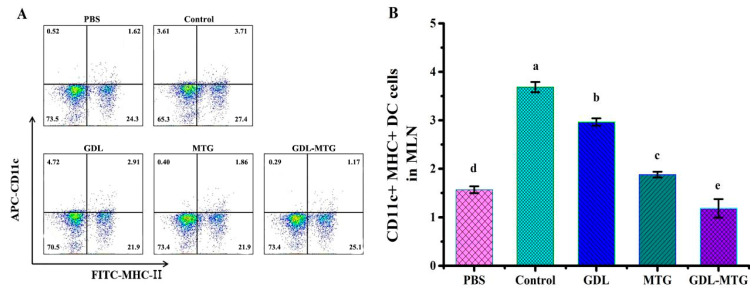
Identification of dendritic cells (DCs) in MLNs: (**A**) Representative dot plots showing percentages of CD11c+ MHC-II+ cells in MLNs. (**B**) Percentages of CD11c+ MHC-II+ cells from the MLNs. Control: soybean protein; GDL: gluconic acid lactone tofu; MTG: microbial transglutaminase-cross-linked tofu; GDL−MTG: composite coagulant tofu. Different letters indicate statistically significant differences (*p* < 0.05).

Dendritic cells (DCs) are specialized antigen-presenting cells that can activate T cells and are central to initiating, regulating, and maintaining the immune response. The main characteristic surface marker of mature DCs is CD11c, so the expression of DC cells was detected by CD11c+ MHC-II+ (Figure 3). In comparison with the PBS group, the expression of DC was significantly higher in the control and GDL groups, whereas the expression of DC in the MTG group was higher than that in the PBS group but lower than that in the control and GDL groups. In particular, the expression of DCs in the GDL−MTG group was the lowest and there was a significant difference between all groups (*p* < 0.05), indicating that adding MTG to tofu can inhibit the activation of DCs.

### 3.3. Detection of CD4/CD8 Cells

**Figure 4:**
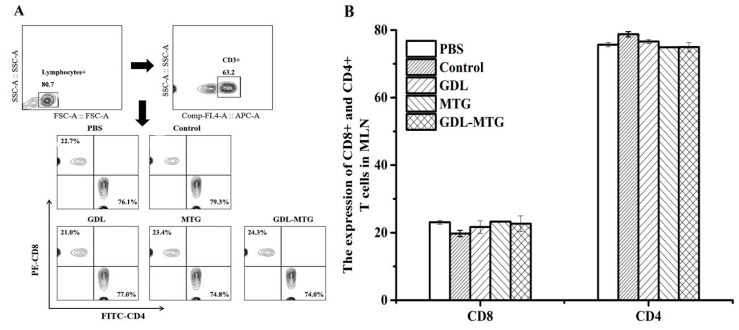
Differentiation of balance on CD4+ and CD8+ T lymphocyte subpopulations from mouse MLNs (**A**) and percentages of CD4+ and CD8+ T cells from the MLNs (**B**). Control: soybean protein; GDL: gluconic acid lactone tofu; MTG: microbial transglutaminase-cross-linked tofu; GDL−MTG: composite coagulant tofu.

Antigens are taken up and processed by DCs and presented to T cells in the form of antigenic peptide–MHC complexes, which in turn trigger a cellular immune response. Mature T cells generally express only CD4 or CD8 molecules, such as CD4+ T cells and CD8+ T cells. Figure 4 shows the differentiation of mouse MLN T lymphocytes CD4+ and CD8+. From this figure, we found that the expression of CD4+ T cells reached more than 70%, and the expression of CD8+ T cells was more than 20%. Meanwhile, the expression was stable in all groups of mice, and there was no significant difference between groups.

### 3.4. Identification of T Lymphocyte Subsets

**Figure 5:**
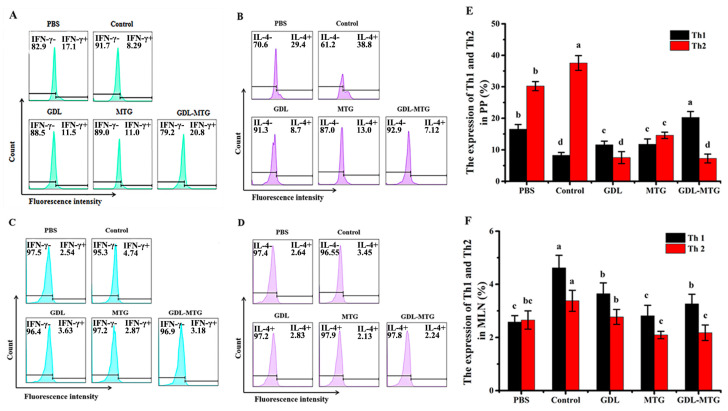
Differentiation balance of Th1/Th2 in PP and MLN: (**A**,**B**) Representative histogram showing the expression of Th1 and Th2 subpopulations in PPs. (**C**,**D**) Representative histogram showing the expression of Th1 andTh2 subpopulations in MLNs. (**E**,**F**) Percentage of Th1 and Th2 subpopulations from the PPs and MLNs, respectively. Control: soybean protein; GDL: gluconic acid lactone tofu; MTG: microbial transglutaminase-cross-linked tofu; GDL−MTG: composite coagulant tofu. Different letters indicate statistically significant differences (*p* < 0.05).

CD4+ T cells could differentiate into T helper (Th) cells upon stimulation, and the precursor cells of Th further differentiate into Th1 and Th2 cells. Studies have shown that allergic reactions are associated with the dysregulation of allergen-specific Th1 and Th2 cell homeostasis, manifested by an over-reactivity of Th2 cells [27,28]. Therefore, we determined the expression of Th1/Th2 in the PPs and MLNs of mice (Figure 5). In comparison with the PBS group, the percentage of Th1 was significantly lower in the PPs of mice in the soybean group (control), while the expression in the GDL and MTG groups was lower than that in the PBS group but higher than that in the soybean group, and there were significant differences. In comparison with the soybean group (control), the percentage of Th2 was significantly lower in the tofu group, and there were significant differences. The expression of Th1 and Th2 in MLNs was the same as in PPs, which showed a significant increase in the Th1 cell population and decrease in the Th2 cell population. So, these results indicated that MTG cross-linked tofu induced a shift in the Th1/Th2 balance toward to Th1 in mice, and alleviated the allergic response.

**Figure 6:**
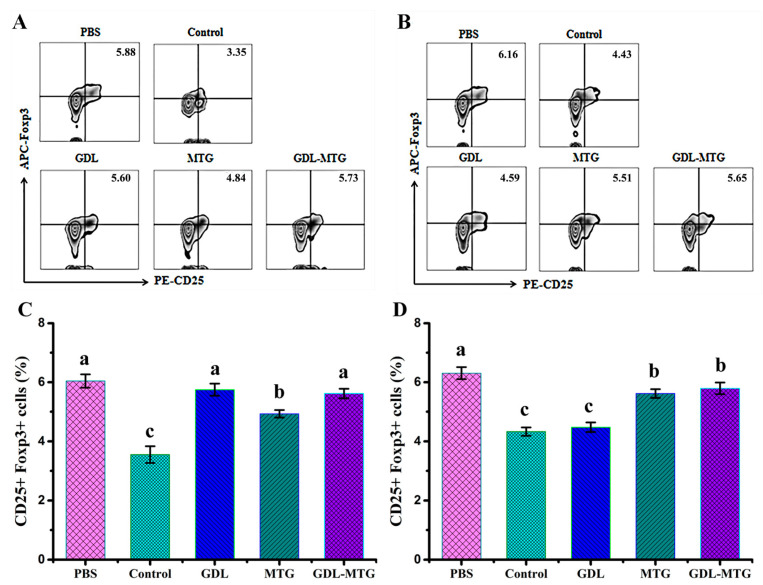
Identification of regulatory T cells (Tregs) in PPs and MLNs: (**A**,**B**) CD25+Foxp3+ expression of Tregs and (**C**,**D**) percentage of CD25+Foxp3+ in CD4+ cell population from PPs and MLNs, respectively. Control: soybean protein; GDL: gluconic acid lactone tofu; MTG: microbial transglutaminase-cross-linked tofu; GDL−MTG: composite coagulant tofu. Different letters indicate statistically significant differences (*p* < 0.05).

Figure 6 shows the percentage of CD25+Foxp3+ in the CD4+ T cell population in the PPs and MLNs of mice identified by flow cytometry. It was found that the expression of CD25+Foxp3+ in mice in the PBS group was the highest, with 5.88% and 6.47% in PPs and MLNs, respectively. In comparison with the PBS group, the percentage of CD25+Foxp3+ in mice in the control group was reduced by 43.0% and 13.9%, respectively, while the other three groups showed an increase in CD25+Foxp3+, and all these results indicated that soybean allergy belongs to the Th2-type allergic reactions. The allergic reaction in the MTG and GDL−MTG groups of the mice was significantly attenuated and tended to be tolerated. These results also suggested that using MTG as a coagulant could shift the Th1/Th2 balance toward to Th1 by regulating Treg levels.

### 3.5. Histopathological Section of the Intestine

**Figure 7:**
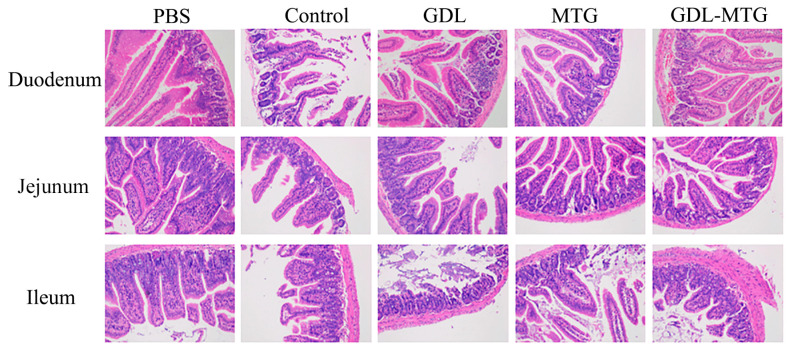
Histological sections (HE) of intestine tissues from the mice (200×). Control: soybean protein; GDL: gluconic acid lactone tofu; MTG: microbial transglutaminase-cross-linked tofu; GDL−MTG: composite coagulant tofu.

The HE staining of each intestinal segment of the small intestine is shown in Figure 7. In comparison with the PBS group, severe atrophy and fracture of intestinal villi and necrosis and detachment of villi epithelium were observed in the duodenum, jejunum, and ileum of mice in the soybean group (control), while less obvious symptoms were shown in the enzyme-cross-linked tofu group. Although there was a certain degree of intestinal villi atrophy, the symptoms were milder than those in the normal tofu group.

## 4. Discussion

Although there are significant differences in allergic reactions between humans and mice [29], mouse models are commonly used to investigate the mechanism of allergic reactions [30]. Because of their high susceptibility and remarkable hypersensitization reactions to food allergens, the BALB/c mice are a typical strain used to study food allergy [31,32,33]. In this study, the allergic symptom scores and serum IgE levels were significantly increased, but the body weight and temperature were significantly decreased in the soy protein group (control) in comparison with the PBS group. These results demonstrated that the soybean-induced allergy mouse model was established successfully (Figure 2). Similarly, in previous studies, the BALB/c mice also displayed obvious humoral immune reactions, cellular immune responses, and systemic allergy symptoms through oral administration of cow milk [34], peanut [35], egg [36], wheat [37], and fish [38] allergens combined with cholera toxin (CT). Compared with the soy protein group (control), the body weight and temperature were increased, but the allergic symptom scores were decreased in the MTG and GDL−MTG groups, with no significant differences compared to the PBS group. These results illustrated that MTG cross-linking could reduce the sensitization to tofu. Therefore, the effect of enzyme-cross-linked tofu on intestinal mucosal immunity was further assessed.

It has been reported that DCs distributed in the intestinal lamina, pooled lymph nodes, and mesenteric lymph nodes play an important role in regulating the immune response of Th2 cells in food allergy. Moreover, DCs, specialized antigen-presenting cells (APCs), are also the only APCs capable of activating the initial T cells. In addition to antigen presentation, DCs play an important role in the regulation of the immune response through the interaction of co-stimulatory molecules. Studies have used mouse models to examine particular sub-populations of DCs activated in food allergies. A study showed that both CD11b cDCs and CD103 cDCs in the MLNs of mice were significantly elevated when reactions were induced by peanut with CT [39]. Another study also illustrated that mice orally sensitized with ovalbumin and CT had increased total DC numbers in the MLNs [40]. In our study, the levels of DCs in the MLNs of mice fed with soy protein were also higher than those in the mice fed with PBS. This result revealed that intake of soy allergy protein could promote the activation of DCs in mouse MLNs. Previous studies have shown that oral administration of highly nitrated OVA induced a regulated DC phenotype, including a decrease in the activation marker of CD86, and an increase in the memory Tregs and IL-10 levels [41]. In a mouse experiment, dietary interventions in combination with galactosaccharides and *Bifidobacterium brachyceae* inhibited the activation of DCs in the intrinsic layer of the small intestine and restored phagocytosis and CD103+ expression to normal levels [42]. Our experimental results are consistent with the above findings, showing that the levels of DCs in the MLNs of mice fed with enzyme-cross-linked tofu are lower than those of mice fed with soy protein, suggesting that their antigen uptake and presentation capacity are lower than those of the soy protein group (control).

The gut-associated lymphoid tissue (GALT) is the lymphoid tissue located under the intestinal mucosa. It is mainly composed of small intestinal Peyer’s patches (PPs), intestinal isolated lymphoid follicles, and mesenteric lymph nodes (MLNs), which are the site of antigen recognition and activation of intestinal mucosal immune cells [43]. Antigen-loaded DC migrated to the MLNs through afferent lymphatics, and then the antigens were presented to naive T cells and induced naive T cells to differentiate into Th1, Th2, and Th17 effector cells [44]. Studies have shown that allergic patients have increased numbers of responding partial T cells and expressed high levels of Th2-type cytokines and receptors, with a hyper-reactive state of Th2 cells [45]. Similarly, our study also showed that the number of Th2-type cells was significantly increased, while the proportion of Th1-type cells was decreased in the lymphoid tissues of allergic mice compared with non-allergic mice (PBS group) (Figure 5). The differentiation and reduced function of Treg removes constraints on the function of Th1, Th2, and Th17 effector cells, thereby inducing a protective immune response against the allergen. IL-10 produced by Treg acts as a check and balance on the activation and function of Th1 and Th2. Meanwhile, it has a strong ability to regulate the inflammatory response in the intestine. It has been shown that increased levels of Tregs in a mouse model could inhibit inflammation of the intestine [46,47]. In recent years, it has been shown that healthy people were more tolerant to allergens than allergic patients, mainly due to the presence of higher numbers of regulatory T cells, and that these could induce peripheral immune tolerance against self- and foreign antigens and suppress allergic reactions [28,48]. Our study also reached a similar conclusion that the mice fed with MTG-cross-linked tofu could promote the differentiation of Th0 cells into Th1 and Treg cells, and inhibit the proportion of Th2 cells, thus alleviating the occurrence of allergic reactions (Figure 6). Collectively, all the results mentioned above showed that in comparison with the soy protein group mice (control), the proportion of Th2 cells was reduced and the proportion of Th1 and Treg cells was increased in the PPs and MLNs of mice fed with enzyme cross-linked tofu, which illustrated that the increase in Tregs shifted the Th1/Th2 balance toward to Th1.

An increase in intestinal permeability has been associated with food allergy and anaphylactic susceptibility [49]. Some studies showed that the allergic response led to intestinal damage, such as the rupture of intestinal villi and an increase in intestinal permeability and the numbers of apoptotic cells [50,51]. In our study, it was shown that the mice in the soy protein group (control) had severe intestinal damage, such as intestinal villus fracture, shedding, and so on, but a milder degree of lesions was observed in the intestine of mice fed enzyme cross-linked tofu (Figure 7). Meanwhile, the increased intestinal permeability could promote allergens passing through the intestinal barrier as well as increasing antigen exposure, activating the submucosal immune system and ultimately leading to an allergic response [52]. This was also confirmed by our study, where after MTG cross-linking, tofu was able to regulate cellular immunity, such as by increasing the level of Tregs to shift the Th1/Th2 balance toward Th 1 (Figure 5).

## 5. Conclusions

In summary, the BALB/c mouse model was used to study the effect of MTG-cross-linked tofu on the intestinal mucosa immunity. The results revealed that the immune system of the intestinal mucosa had a low sensitivity to MTG-cross-linked tofu, mainly manifested by a decrease in dendritic cell (DC) levels, an increase in Th1 and Treg cells, and a decrease in Th2 cells of the PPs and MLNs. Our results will provide a theoretical basis for the development of enzyme-cross-linked hypoallergenic soy products.

## Figures and Tables

**Table 1:** Anaphylactic symptom scoring.

Score	Symptoms
0	No symptoms
1	Scratching nose and mouth
2	Swelling around the eyes and mouth; pillar erection; reduced activity; higher breathing rate
3	Shortness of breath; blue rash around the mouth and tail; higher breathing rate
4	No activity after stimulation; shivering and muscle contractions
5	Death by shock

## Data Availability

The original contributions presented in the study are included in the article, further inquiries can be directed to the corresponding author.
